# Enzyme classification with peptide programs: a comparative study

**DOI:** 10.1186/1471-2105-10-231

**Published:** 2009-07-24

**Authors:** Daniel Faria, António EN Ferreira, André O Falcão

**Affiliations:** 1 Department of Informatics, Faculty of Sciences, University of Lisbon, 1749-016 Lisbon, Portugal; 2 Center of Chemistry and Biochemistry, Department of Chemistry and Biochemistry, Faculty of Sciences, University of Lisbon, 1749-016 Lisbon, Portugal

## Abstract

**Background:**

Efficient and accurate prediction of protein function from sequence is one of the standing problems in Biology. The generalised use of sequence alignments for inferring function promotes the propagation of errors, and there are limits to its applicability. Several machine learning methods have been applied to predict protein function, but they lose much of the information encoded by protein sequences because they need to transform them to obtain data of fixed length.

**Results:**

We have developed a machine learning methodology, called peptide programs (PPs), to deal directly with protein sequences and compared its performance with that of Support Vector Machines (SVMs) and BLAST in detailed enzyme classification tasks. Overall, the PPs and SVMs had a similar performance in terms of Matthews Correlation Coefficient, but the PPs had generally a higher precision. BLAST performed globally better than both methodologies, but the PPs had better results than BLAST and SVMs for the smaller datasets.

**Conclusion:**

The higher precision of the PPs in comparison to the SVMs suggests that dealing with sequences is advantageous for detailed protein classification, as precision is essential to avoid annotation errors. The fact that the PPs performed better than BLAST for the smaller datasets demonstrates the potential of the methodology, but the drop in performance observed for the larger datasets indicates that further development is required.

Possible strategies to address this issue include partitioning the datasets into smaller subsets and training individual PPs for each subset, or training several PPs for each dataset and combining them using a bagging strategy.

## Background

Reliable automated protein function prediction is one of the main challenges faced by Biology in the post-genomic age, as the gap between gene or protein sequences and experimentally determined functional annotations continues to increase.

For over a decade, biologists have relied on alignment algorithms such as BLAST [1] for function prediction, under the assumption that proteins of similar sequence should perform the same function(s) [2]. However, this assumption is only safe for high levels of sequence similarity and even then detailed function predictions are not always accurate [2,3]. Furthermore, the pervasive use of this alignment-based approach promotes the propagation of errors, as the function of new proteins is inferred from proteins whose function was already inferred, in a chain that leads to a small fraction of proteins with experimentally determined function [4,5].

As an alternative to the alignment-based approach, several machine learning methods have been proposed for protein function prediction/classification [6]. Methods such as decision trees [7,8], neural networks [9,10] and more prominently support vector machines (SVMs) [11-19] have all been applied to specific prediction/classification tasks with some success. These methods are theoretically less prone to propagate errors than the alignment-based approach, because they classify new proteins based on models learned from large sets of proteins rather than by direct inference based on a few proteins. Furthermore, some of these methods have performed well in cases of low sequence similarity, which suggests they could complement the alignment-based approach [6]. Another interesting advantage of machine learning methods is that several classifiers can be combined to form ensembles, which have an increased performance when compared with the best individual classifiers [20].

However, most machine learning methods share one shortcoming regarding protein classification: they were designed to deal with data of fixed length and thus require protein sequences to be transformed into vectors of (sequence-derived) features [21]. While several efforts have been made to encode as much information about the sequence as possible in these feature vectors [21-24], there is always a loss of information associated with the transformation. Thus, a machine learning method suited to deal directly with sequence data could be advantageous for protein classification, particularly for detailed classification tasks.

We have previously conceived a novel machine learning methodology, called Peptide Program (PP), which was designed to deal directly with protein sequences [25]. The concept behind this methodology is that a protein can be represented by a very simple computer system: each of the twenty amino acids is assigned a small computer program, and those programs are executed according to the sequence of the protein, changing the state of the system. A PP can be used for classification by selecting a set of programs for the amino acids such that, for proteins that belong to a target class, the final state of the system is distinguishable from those that don't.

In this work, after further development of the PP methodology, we compare its performance with that of SVMs and BLAST in detailed enzyme classification tasks.

## Results and discussion

### Classification Tasks

The PP methodology was evaluated in eighteen binary classification tasks, consisting in identifying proteins that belong to one of eighteen selected Enzyme Commission (EC) families (e.g. EC 1.1.1.1, EC 2.7.2.1) from among all those that belong to the respective EC sub-subclass (e.g. EC 1.1.1.-, EC 2.7.2.-). This type of detailed classification task was chosen because it is where the advantages of dealing directly with sequences over dealing with sequence-derived features are more likely to be felt. The assumption is that identifying the EC sub-subclass is a more trivial task, whereas identifying the EC family after knowing the EC sub-subclass will pose some challenges because the sequence similarity between the negatives and positives is expected to be fairly high.

The eighteen EC families selected were well represented (including around 200 proteins each) and covered all six major EC classes (i.e. EC 1. to 6.). 20% of the positives and negatives for each family were separated for testing and the remaining 80% were used to train the classifiers, or as database in the case of BLAST.

The families selected and the size of the positive and negative training and testing sets are shown in Table 1. The average sequence identity between each positive test protein and its closest positive training protein (SICP) is high for all EC families (>70%), but for most families there is also significant average identity (>30%) between positive test proteins and the closest negative training proteins (SICN). Furthermore, there are several cases of test proteins that are nearly as similar or more similar to train proteins of the opposite class (Table 1).

**Table 1:** Characterization of the EC families selected

EC family	P Train	N Train	P Test	N Test	Avg SICP	Std SICP	Avg SICN	Std SICN	NNP	NPN
1.1.1.1	168	3611	42	893	85%	15%	47%	9%	2	9
1.1.1.25	173	3607	44	890	79%	21%	41%	6%	6	4
1.8.4.11	163	156	41	40	87%	15%	18%	24%	2	5
2.1.2.10	172	1300	44	325	86%	16%	26%	17%	2	0
2.3.2.6	160	160	41	41	82%	18%	31%	21%	2	1
2.5.1.55	161	2652	41	648	94%	8%	41%	12%	1	1
2.7.1.11	162	3381	41	844	86%	20%	46%	5%	4	1
2.7.1.21	165	3381	42	840	83%	21%	39%	17%	7	1
2.7.2.1	173	942	44	236	85%	15%	46%	4%	1	1
2.7.7.27	167	5606	42	1313	89%	13%	29%	3%	0	4
3.1.26.11	168	1109	42	278	85%	15%	24%	17%	1	3
3.5.4.19	175	1549	44	388	84%	14%	30%	21%	0	0
4.1.1.31	161	2603	41	649	87%	17%	18%	18%	1	1
4.2.3.4	172	488	43	123	79%	20%	23%	21%	3	0
5.1.1.1	175	387	44	97	79%	23%	20%	17%	3	2
5.1.1.3	163	400	41	99	88%	17%	30%	17%	2	2
5.3.1.24	173	1744	44	431	78%	20%	43%	14%	8	3
6.3.4.3	164	1071	42	267	84%	15%	31%	14%	0	0

P Train – size of positive training; N Train – negative training; P Test – positive testing; N Test – negative testing; Avg SICP – average sequence identity between each positive test protein and the closest positive training protein; Std SICP – standard deviation of the SICP; Avg SICN – average sequence identity between each positive test protein and the closest negative training protein; Std SICN – standard deviation of the SICN; NNP – near negative positives (i.e. positive test proteins that have a closest negative at least within 10% sequence indentity of the closest positive); NPN – near positive negatives.

### Peptide Programs Results

Overall, the performance of the PP methodology was good, with an average Matthews Correlation Coefficient (MCC) in testing of 0.95, an average precision of 98% and an average recall of 94% (Table 2). Five of the EC families tested were perfectly classified (MCC of 1) and only five had MCC below 0.95.

**Table 2:** Training and test results for EC family classification using Peptide Programs

	Training				Test			
	
EC Family	Accuracy	Precision	Recall	MCC	Accuracy	Precision	Recall	MCC
1.1.1.1	99%	97%	87%	0,91	99%	94%	81%	0.87
1.1.1.25	100%	99%	94%	0,96	99%	93%	84%	0.88
1.8.4.11	100%	100%	100%	1,00	100%	100%	100%	1.00
2.1.2.10	100%	100%	100%	1,00	100%	98%	100%	0.99
2.3.2.6	100%	100%	100%	1,00	100%	100%	100%	1.00
2.5.1.55	100%	100%	100%	1,00	100%	98%	98%	0.97
2.7.1.11	100%	100%	96%	0,98	100%	100%	90%	0.95
2.7.1.21	99%	99%	86%	0,92	99%	97%	79%	0.87
2.7.2.1	100%	100%	100%	1,00	100%	100%	100%	1.00
2.7.7.27	100%	96%	93%	0,95	99%	93%	88%	0.90
3.1.26.11	100%	100%	99%	1,00	99%	93%	100%	0.96
3.5.4.19	100%	100%	97%	0,98	99%	100%	91%	0.95
4.1.1.31	100%	100%	100%	1,00	100%	98%	100%	0.99
4.2.3.4	100%	100%	100%	1,00	99%	100%	98%	0.98
5.1.1.1	100%	100%	100%	1,00	100%	100%	100%	1.00
5.1.1.3	100%	100%	100%	1,00	100%	100%	100%	1.00
5.3.1.24	99%	100%	93%	0,96	98%	95%	82%	0.87
6.3.4.3	100%	100%	100%	1,00	99%	98%	98%	0.97
Average	100%	100%	97%	0,98	99%	98%	94%	0.95

All results were obtained with PP classifiers with 4 registers and 2 instructions per amino acid, except those of families 1.1.1.1 (which had 6 registers) and 1.8.4.11 (which had 2 registers and 1 instruction per amino acid).

Notably, the worse results occurred for EC families with large datasets, and there was a negative correlation between the size of the datasets and the quality of the results in terms of MCC (correlation coefficient of -0.73 for testing results and -0.72 for training results). Thus, while a PP is able to encode specific sequence patterns that differentiate a group of sequences from another, it has more difficulty when those groups are larger, which is only natural, as it is generally harder to learn a model that satisfies a larger and more diverse dataset.

It is interesting to note that the drop in performance was felt mainly in terms of recall, which decreased 11% between the nine EC families with the smallest datasets and those with the largest (see Table 1). By contrast, the precision dropped only by 3%. This is indicative of the sequence specificity of the PP methodology, as the sequence patterns it encodes are able to exclude the negative test proteins, even when they don't model the training data perfectly.

There was only a very weak correlation between the performance of the PPs and SICP (correlation coefficient of 0.24) and a mild correlation with SICN (correlation coefficient of -0.56). This suggests that although the PP methodology is influenced by sequence similarity, it is not directly dependent upon it to encode class discriminating sequence patterns. Further evidence of this is given by the fact that the PPs were able to classify correctly 71 of the 83 testing proteins which were nearly as similar or more similar to training proteins of the opposite class than to proteins of their class (near positives and near negatives).

### Peptide Program Configuration

The PP framework includes several parameters which can be altered: the number of registers (which store the state of the system), the number of instructions per amino acid, the limit value of the registers, the threshold value used to compare registers, and the maximum value that can be added to or subtracted from a register. Of these parameters, the number of registers and to a lesser degree the number of instructions per amino acid were found to have the strongest influence on the performance of the methodology, because they define the amount of information a PP can encode and consequently, its complexity. On one hand, a simpler PP can't encode as much information as a more complex one and will have more difficulty in modelling large and/or diverse groups of proteins; on the other hand, the more complex the configuration, the larger the search space of PP solutions, and the more difficult it will be to train a PP to model any group of proteins.

An intermediate configuration with 4 registers and 2 instructions per amino acid was empirically determined to be the best for the dimension of the classification problems in this work, and was initially used for all EC families. Afterwards, a more complex configuration with 6 registers (and also 2 instructions per amino acid) was tested for the families with the larger datasets, as the intermediate configuration wasn't able to model these perfectly. However, the more complex configuration only produced better results than the intermediate configuration for family EC 1.1.1.1. Likewise, a simpler configuration of 2 registers and 1 instruction per amino acid was tested for the families with the smaller datasets, but performed worse than the intermediate configuration for all families except family EC 1.8.4.11, where both configurations produced perfect classifiers. Thus, the intermediate configuration was indeed the best overall.

### Support Vector Machine Results

The performance of the SVMs was similar to that of the PPs, with a slightly higher average MCC in testing of 0.96, a lower average precision of 96%, a higher average recall of 97%, and seven perfectly classified EC families (Table 3). In contrast with the PPs, all EC families were perfectly modelled in training with SVMs, and there was only a small negative correlation between SVM performance and the size of the datasets (correlation coefficient of -0.33). This decrease in performance was observed both in terms of precision and recall, with the former dropping by 6% and the latter by 4% from the nine smallest to the nine largest datasets. However, the precision was generally lower than the recall, independently of the size of the datasets.

**Table 3:** Test results for EC family classification using Support Vector Machines

EC family	Accuracy	Precision	Recall	MCC
1.1.1.1	98%	80%	86%	0.82
1.1.1.25	99%	93%	91%	0.92
1.8.4.11	100%	100%	100%	1.00
2.1.2.10	100%	100%	100%	1.00
2.3.2.6	100%	100%	100%	1.00
2.5.1.55	100%	100%	100%	1.00
2.7.1.11	100%	98%	98%	0.97
2.7.1.21	99%	93%	88%	0.90
2.7.2.1	99%	96%	100%	0.97
2.7.7.27	100%	100%	100%	1.00
3.1.26.11	99%	98%	98%	0.97
3.5.4.19	99%	96%	98%	0.96
4.1.1.31	100%	95%	100%	0.96
4.2.3.4	99%	100%	95%	0.97
5.1.1.1	100%	100%	100%	1.00
5.1.1.3	97%	95%	95%	0.93
5.3.1.24	98%	86%	95%	0.89
6.3.4.3	100%	100%	100%	1.00
Average	99%	96%	97%	0.96

All results were obtained with SVMs using polynomial kernels, except for families 2.5.1.55 and 2.7.7.27, which were obtained with radial basis kernels. The training results were perfect for all EC families.

These differences are likely related to the fact that SVMs deal with derived global sequence features and thus do not have the sequence specificity of the PPs. On one hand, SVM deal more easily with large datasets, because the variability of the global sequence features is less than the variability of the sequences. On the other hand, because they lack the sequence specificity of the PPs, and since the number and diversity of the negatives are greater than the number and diversity of the positives, it is only natural that the SVMs misclassify more negatives than positives, and thus have a lower precision than recall.

The correlations between SVMs and SICP, and between SVMs and SICN (correlation coefficients 0.27 and -0.55 respectively) were surprisingly similar to those observed for the PPs. Since the SVMs do not deal directly with sequences, we would expect their dependency upon sequence similarity to be smaller than that of the PPs, however, that appears not to be the case. Nevertheless, the SVMs did perform better than the PPs regarding the near positives and near negatives, classifying correctly 75 out of 83 of these.

The Q-statistic, which provides a measure of similarity between classifiers [26], had a value of 0.98 between PPs and SVMs. This value is deceivingly high, considering that less than 1% of the proteins were misclassified by either methodology. Indeed, considering only the misclassified proteins, the two methodologies agreed only on 10% of the cases. Thus, the two methodologies err mostly on different cases.

### BLAST Classifier Results

The results of the BLAST classifiers were better than both PPs and SVMs, with an average MCC of 0.98, an average precision of 99%, and an average recall of 98% (Table 4). This is not surprising since sequence similarity has likely been behind many of the assignments of proteins to EC families, as evidenced by the high average SICP observed for all EC families tested. Furthermore, the classification tasks are simpler for BLAST than for the PPs and SVMs, as these have to model each EC family globally whereas BLAST only considers the local neighbourhood of each protein.

**Table 4:** Test results for EC family classification using BLAST

EC family	Accuracy	Precision	Recall	MCC
1.1.1.1	100%	95%	98%	0.96
1.1.1.25	100%	100%	98%	0.99
1.8.4.11	98%	98%	98%	0.95
2.1.2.10	100%	100%	98%	0.99
2.3.2.6	100%	100%	100%	1.00
2.5.1.55	100%	100%	100%	1.00
2.7.1.11	100%	100%	98%	0.99
2.7.1.21	99%	97%	90%	0.94
2.7.2.1	100%	100%	100%	1.00
2.7.7.27	100%	100%	100%	1.00
3.1.26.11	100%	100%	98%	0.99
3.5.4.19	100%	100%	100%	1.00
4.1.1.31	100%	100%	100%	1.00
4.2.3.4	99%	100%	95%	0.97
5.1.1.1	96%	100%	89%	0.92
5.1.1.3	99%	98%	100%	0.98
5.3.1.24	100%	96%	100%	0.98
6.3.4.3	100%	100%	100%	1.00
Average	99%	99%	98%	0.98

Thus, it is interesting to note that the PP methodology performed slightly better than both BLAST and SVMs for the smaller datasets, and had a global precision nearly as high as BLAST, despite the loss in recall for the larger datasets. This is indicative of the discriminating power of the PP methodology, and of its potential for detailed classification tasks.

As would be expected, the BLAST classifiers performed worse than the PPs and SVMs regarding the near positives and near negatives, classifying correctly 65 out of 83 of these proteins. Notably, out of the 24 proteins misclassified with BLAST (either near positives/negatives or below the sequence similarity threshold considered), the PPs were able to correctly classify 16, and the SVMs were able to classify 15. This reinforces the fact that the PP methodology is able to capture discriminatory sequence patterns independently of sequence similarity.

The Q-statistic between PPs and BLAST was 0.98 and between SVMs and BLAST was 0.99. Similar to what was observed between PPs and SVMs, the former value corresponds to 8% agreement regarding misclassified proteins, whereas the latter corresponds to 11% agreement. So, overall the three methodologies differ significantly in the errors they make.

## Conclusion

The PP methodology was conceived under the assumption that a machine learning method that dealt directly with sequence data could be advantageous over traditional machine learning methods, as the latter lose much of the information encoded by protein sequences in the transformation into feature vectors.

While the global quality of the PP and SVM classifiers was similar, the PPs had a clear advantage in terms of precision, despite the fact that they were negatively influenced by the size of the datasets in terms of recall. These differences are tied to the fact that variability between proteins is greater in the sequence space than in the SVM feature space: on one hand, it is easier to distinguish between proteins and encode a very restrictive pattern that excludes all negatives; on the other hand, there is much more "noise" (i.e. differences and similarities between proteins with no relevance for their classification), and thus it is harder to encode a pattern that satisfies all positives, particularly for large datasets. Since precision is essential to avoid annotation errors, we can consider that dealing directly with sequences is indeed advantageous for detailed protein classification.

In comparison with BLAST, the PP methodology performed slightly better for the EC families with the smaller datasets and worse for the families with the larger datasets, which reflects the different classification strategies of the two methodologies. The PP methodology has to derive a model to fit all the training proteins, which will be more difficult the greater the size and diversity of the training set. By contrast, the BLAST classifier only requires that each test protein have one or more similar training proteins, and therefore is not directly influenced by the size or diversity of the training set.

One possibility to address this issue would be to partition the EC families according to sequence similarity and train one PP per partition rather than one PP per family, in order to reduce the size and complexity of each classification task. Another possibility would be to train several PP classifiers for each EC family and combine them in a bagging strategy [27], since the loss in performance for the larger datasets is felt mostly in terms of recall.

It is important to note that the PP framework used in this work is only one of many possible implementations of the methodology, and was designed to encode sequence patterns for the purpose of detailed classification. Future work will focus on alternative frameworks, in order to explore the potential of the methodology for more general classification problems and larger datasets. In particular, the use of condensed amino acid alphabets [19,23] will be considered, as will the use of partial sequences, such as the sequence of amino acids occurring in the surface of the protein [28], or of those within a particular secondary structure. Additionally, the fact that there was little overlap between the proteins misclassified with PPs and those misclassified with SVMs suggests that is worth exploring the combination of the two methodologies into an ensemble of classifiers [20].

Another aspect of the PP methodology that is important to mention is that it is computationally very efficient, as it takes under 100 μs to test a protein and requires no pre-processing of protein sequences. This means that it is feasible to use several PP classifiers (nested and/or in parallel) to identify the functional class of new proteins within a computational time that can compete with BLAST.

## Methods

### Dataset

Eighteen families were selected from the ENZYME database [29] under the criteria that each family selected had around two hundred proteins (as of July 2008) and that each of the six major EC classes was represented in the selection. The number two hundred had no particular meaning other than ensuring all classes selected were well represented, whereas limiting our dataset to eighteen classes was simply a compromise between variety and processing time.

The positives for each family (e.g. EC 1.1.1.1) were all proteins belonging to that family, excluding those with partial sequences. The negatives were all proteins which belonged to the same sub-subclass (e.g. EC 1.1.1.-) but not the same family, again excluding those with partial sequences.

20% of the positives and negatives for each family were used for testing and the remaining 80% were used for training.

### Peptide Program Framework

The PP framework was developed from that presented in previous work [25], while retaining the same basic structure:

• Each amino acid is represented by a program consisting of a number of instructions *I*.

• A number of registers *R *record the state of the system, storing values in a range [-*N*, *N*].

• Each instruction comprises a condition and an operation.

• The condition verifies the state of the system by comparing two registers, using the operators >>, ≈ and <<. A threshold value *T *is used in the comparisons, so: *R0 *>> *R1 *means *R0 *> *R1 *+ *T*; *R0 *≈ *R1 *means *R1 *- *T *≤ *R0 *≤ *R1 *+ *T*; and *R0 *<<*R1 *means *R0 *<*R1 *- *T*.

• The operation acts upon the system if the condition is met, changing one register by either adding or subtracting a positive numeric value in a range [1, *S*], or adding or subtracting the value of a register (which can be the register being changed).

• Both condition and operation can also be null. If the condition is null, the operation is always executed, and if the operation is null the whole instruction is null.

• The set of instructions assigned to each amino acid and the set of system parameters used constitute one PP.

• The criterion used for classification is that a protein is considered positive if the value of the first register is greater than zero after executing the PP for the whole length of the protein sequence.

In this work, the values of the system parameters *N*, *T *and *S *were fixed as 128, 4 and 8 respectively, as their influence on the performance of the PP is small. The parameters *I *and *R *were initially fixed as 2 and 4 respectively, which were empirically determined to be optimal values. Later a configuration with *R *= 6 was tested for the EC families with the larger datasets, and a configuration with *I *= 1 and *R *= 2 was tested for the families with the smaller datasets.

### Peptide Program Example

Considering only the amino acids alanine (A), cytosine (C), and glycine (G), a simple PP with 2 registers, 1 instruction per amino acid and a threshold value of 4 could be:

• A: if(Reg[1] ≈ Reg[2]): Reg[2] + = 4

• C: if(Reg[2] >> Reg[1]): Reg[1] + = Reg[2]

• G: Reg[1] - = 3

The execution of this PP for a putative protein sequence ACGACG would be the following:

• At the start both registers have the value 0

• 1^st ^position A: the condition is true, so 4 is added to the second register

• 2^nd ^position C: the condition is false (the difference between registers is within the threshold value) so nothing is done

• 3^rd ^position G: there is no condition, so 3 is subtracted from the first register

• 4^th ^position A: the condition is false so nothing is done

• 5^th ^position C: the condition is true, so the value of the second register, 4, is added to the first register, which now has the value 1

• 6^th ^position G: there is no condition, so 3 is subtracted from the first register, which now has the value -2

• Thus at the end of the protein the first register has the value -2 and the second register has the value 4. Since the first register has is smaller than zero, the protein would be considered negative by this PP, in the classification task it was trained for.

### Peptide Program Training

Each PP was trained using a heuristic optimization algorithm based on simulated annealing [30]:

• There are ten steps of 15000 iterations.

• The first step starts with a random PP, while the subsequent steps start with the best solution obtained thus far.

• The first nine steps are simulated annealing steps, meaning that there is a probability that a worse solution is temporarily accepted. That probability is given by *P *= *exp*(Δ*x*/*T*), where Δ*x *is the difference between the current solution and the previous one and *T *is the current annealing temperature. The last step is a fine-tuning step, so only solutions that are better than or equal to the previous are accepted.

• The annealing steps have an exponential cooling schedule, with a starting temperature such that in the first iteration there is a 10% probability of accepting a solution which is 10% worse; and after 12000 iterations the probability of accepting a solution which is 0.1% worse is only one in a million. During the last 3000 iterations the effect of the temperature is negligible.

• On each iteration, an amino acid and one of its instructions are randomly chosen and that instruction is changed to a new random instruction.

• The PP with the new instruction is evaluated over the training set, using the Matthews Correlation Coefficient (MCC) as the evaluation criterion. It will be accepted if it is better than or equal to the previous solution, or under the annealing probability. If it isn't accepted, the instruction changed reverts to its previous form.

• In addition to the current solution, the system keeps track of the best solution found thus far. At the end of each step, if the current solution is not the best solution, it reverts to the best solution for the start of the next step.

For each EC family ten PPs were trained with the default parametrisation (4 registers and 2 instructions per amino acid) and an additional five PPs were trained with the alternative parametrisation (more complex for large datasets and simpler for small datasets). The PP that produced the best training results was kept, or in the few cases where two PPs produced the same training results, the one that produced the best results in testing was kept.

The source code for the PP methodology is available in the Additional file 1.

### SVM Classifier

The SVM implementation used in this work was SVMlight [31]. Protein sequences were transformed into feature vectors by grouping amino acids according to several physicochemical properties and computing the composition, transition and distribution descriptors. This is a popular strategy for protein classification [12,13,15,16,18,22,23], and was implemented as described in [23]:

• The first 20 features are the amino acid composition of the sequence.

• The following seven properties were considered: hydrophobicity, van der Waals volume, polarity, polarizability, charge, secondary structure and solvent accessibility.

• For each property, the amino acids were divided into three discrete groups, according to their value for that property, or in the case of secondary structure and solvent accessibility, according to their probability of occurrence. Thus, for each property, a sequence of twenty amino acids is transformed into a sequence of three groups.

• For each property, the composition, transition and distribution descriptors are computed from this sequence of three groups. The two former descriptors correspond to 3 features per property each, whereas the latter corresponds to 15 features per property. Thus the feature vector of a sequence comprises a total of 167 features.

For each EC family, four SVMs were trained, each with a different type of kernel (linear, polynomial, radial basis and sigmoid). The default SVMlight parameters were used (since they proved suitable) except the weight of the positive errors relative to the negative, which was set to the ratio between the size of the negative and positive training sets. The SVM with the best training results was kept, or in the few cases where two SVMs produced the same training results, the one that produced the best results in testing was kept.

### BLAST Classifier

Unlike the PP and SVM methodologies, there is no training step for the BLAST classification. Instead, for each EC family, the training set is used as the BLAST database, and the proteins in the test set are classified based on their BLAST results against that database:

• BLAST results were evaluated according to the relative BLAST score, which is computed as the score of the alignment divided by the score of the self-alignment of the query protein. This score measures the quality of the alignment independently of the size of the proteins (unlike the absolute BLAST score) but considering the whole protein sequence rather than just the aligned portion (unlike the sequence identity score).

• A minimum relative BLAST score of 30% was considered as threshold, and alignments below threshold were discarded, since this is a detailed classification task and high sequence similarity values are required for detailed enzyme classification [3].

• For each test protein, a result set was retrieved, consisting of the BLAST result with the highest relative score and all other results with relative score within 10% of the highest.

• If no significant BLAST results were retrieved, the protein was considered as negative by default.

• If 90% or more of the proteins in the result set were of the same class and if that was the class of the test protein, it was considered well classified (either true positive or true negative). Otherwise, it was considered misclassified (either false positive or false negative).

## Authors' contributions

DF implemented and developed the PP methodology, executed all steps of the study, and drafted the manuscript. AOF designed the PP methodology, supervised its development, coordinated the study, and helped to draft the manuscript. All authors participated in the conception and design of the study, and read and approved the final manuscript.

## Supplementary Material

Additional file 1 **Peptide Programs**. Source code for the Peptide Program methodology used in this work. Click here for file
